# A Portable Reverse Transcription Recombinase Polymerase Amplification Assay for Rapid Detection of Foot-and-Mouth Disease Virus

**DOI:** 10.1371/journal.pone.0071642

**Published:** 2013-08-20

**Authors:** Ahmed Abd El Wahed, Ayman El-Deeb, Mohamed El-Tholoth, Hanaa Abd El Kader, Abeer Ahmed, Sayed Hassan, Bernd Hoffmann, Bernd Haas, Mohamed A. Shalaby, Frank T. Hufert, Manfred Weidmann

**Affiliations:** 1 Department of Virology, University Medical Center, Goettingen, Germany; 2 Virology Department, Faculty of Veterinary Medicine, Mansoura University, Mansoura, Egypt; 3 Virology Department, Faculty of Veterinary Medicine, Cairo University, Giza, Egypt; 4 Animal Health Research Institute, Giza, Egypt; 5 Animal Health Research Institute, Faiyum, Egypt; 6 Institute of Diagnostic Virology, Friedrich-Loeffler-Institute, Greifswald-Insel Riems, Germany; Virginia Polytechnic Institute and State University, United States of America

## Abstract

Foot-and-mouth disease (FMD) is a trans-boundary viral disease of livestock, which causes huge economic losses and constitutes a serious infectious threat for livestock farming worldwide. Early diagnosis of FMD helps to diminish its impact by adequate outbreak management. In this study, we describe the development of a real-time reverse transcription recombinase polymerase amplification (RT-RPA) assay for the detection of FMD virus (FMDV). The FMDV RT-RPA design targeted the 3D gene of FMDV and a 260 nt molecular RNA standard was used for assay validation. The RT-RPA assay was fast (4–10 minutes) and the analytical sensitivity was determined at 1436 RNA molecules detected by probit regression analysis. The FMDV RT-RPA assay detected RNA prepared from all seven FMDV serotypes but did not detect classical swine fever virus or swine vesicular disease virus. The FMDV RT-RPA assay was used in the field during the recent FMD outbreak in Egypt. In clinical samples, reverse transcription polymerase chain reaction (RT-PCR) and RT-RPA showed a diagnostic sensitivity of 100% and 98%, respectively. In conclusion, FMDV RT-RPA was quicker and much easier to handle in the field than real-time RT-PCR. Thus RT-RPA could be easily implemented to perform diagnostics at quarantine stations or farms for rapid spot-of-infection detection.

## Introduction

FMD is a contagious trans-boundary disease infecting cloven-hoofed animals and leads to huge economic losses (death of young ruminants, diminishes milk, and meat production) [1]. FMDV is a non-enveloped, positive sense single stranded RNA virus belonging to the genus *Aphthovirus* of the *Picornaviridae* family [2]. It has seven serotypes (A, O, C, SAT 1-3, and Asia1) that have a distinct geographical distribution (A and O are widely distributed across the world, SAT 1-3 mainly in Africa and Asia 1 in Asia) [3]. Europe and North America are free of FMDV. Nevertheless, to date no country is considered safe [4]. There is always a fear of introducing FMDV into a FMDV-free country or a new serotype into a FMDV-endemic country. For example serotype O was endemic in Egypt since 1960 [5], and in 2006, type A was introduced and caused a FMD outbreak [6]. Recently, SAT 2 was the primary cause of a FMD epidemic in Egypt which erupted in February 2012 and led to 82362 suspected cases, of which 19655 died [7]. Outbreaks due to SAT 2 were also reported from Libya and the Gaza strip [8], [9]. It is assumed that FMDV SAT2 was introduced from sub-Saharan Africa where it is endemic [9].

FMDV is highly contagious due to the ability of the causative agent to gain entry and initiate infection via a variety of sites, the small infective dose, the short incubation period, and the release of FMDV before the onset of clinical signs. In addition, the massive quantities of virus excreted from infected animals, its ability to spread large distances due to airborne dispersal and the survivability of the virus in the environment contribute to its contagiousness [10]. It is therefore absolutely necessary to detect a FMD outbreaks as early as possible to initiate the appropriate control measures and prevent further spread among livestock. As other diseases may cause clinical signs resembling FMD, often a laboratory confirmation of suspect cases is indispensible. The classical method, virus isolation takes several days and is only possible in a few specialized laboratories. Lateral flow assays [11] and antigen ELISA have a limited sensitivity and yield positive results only with vesicular material but not with saliva, nasal swabs or serum [4]. Currently, laboratory diagnosis of FMD mostly depends on the detection of viral RNA by reverse transcription polymerase chain reaction (RT-PCR) [12]–[14]. Samples collected from animals in the field or at quarantine stations are sent to central laboratories for testing, as RT-PCR assays are not suitable for on-site screening. Therefore, portable, accurate, simple, and rapid tests are needed to detect the virus at the spot-of-infection. Recombinase polymerase amplification (RPA) is an isothermal DNA amplification and detection method [15]. The amplification depends on a specific combination of enzymes and proteins (recombinase, single strand binding protein, and strand displacing DNA polymerase) used at a constant temperature. Real-time detection of RPA amplicons is possible *via* exo-probes. Development of fluorescence depends on the separation of fluorophore and quencher via Exonuclease III cleaving at an internal abasic site mimic (tetrahydrofuran, THF) of the hybridized exo-probe [16], [17]. The fluorescence signal is measured in real-time via a simple point-of-care scanner weighing 1.2 kg including the laptop (ESEQuant Tubescanner device, Qiagen Lake Constance GmbH, Stockach, Germany). This study describes the development of a real-time reverse transcription RPA (RT-RPA) assay for the detection of all FMDV serotypes and its use in the 2012 FMD outbreak in Egypt.

## Materials and Methods

### Ethics Statement

Twenty-seven samples (Table 1) were derived from two animal trial (#FLI 007/08 and #FLI 002/11) performed at the Friedrich-Loeffler-Institute, Greifswald-Insel Riems, Germany. The two experiments were licensed by the Landesamt für Landwirtschaft, Lebensmittelsicherheit und Fischerei Mecklenburg-Vorpommern, Thierfelder Str. 18, 18059 Rostock, Germany with following animal welfare license numbers LALLF M-V/TSD/7221.3-1.1-017/08 and LALLF 7221.3-2.5-002/11.

**Table 1:** Detection of FMDV in experimentally infected animals using real-time RT-PCR and RT-RPA.

Animal ID	Species	Dpi	Sample type	Detection	
				RT-PCR (CT)	RT-RPA (minute)
P23	Pig^1^	3	Serum	29.37	neg
P24	Pig^1^	3	Serum	15.02	3.7
G50	Goat^1^	3	Serum	31.1	neg
G51	Goat^1^	5	Serum	22.55	5.3
S8	Sheep^1^	3	Blood	36.14	neg
S11	Sheep^1^	5	Blood	29.34	6.7
WB18	Wild boar^2^	5	Saliva	30.3	neg
WB18	Wild boar^2^	3	Nasal swabs	29.36	neg
WB18	Wild boar^2^	3	Serum	19.79	5.7
WB18	Wild boar^2^	4	Nasal swabs	26.31	6
WB18	Wild boar^2^	12	Vesicular material	23.71	6
WB19	Wild boar^2^	4	Nasal swabs	24.48	5.3
WB19	Wild boar^2^	8	Saliva	28.88	neg
WB19	Wild boar^2^	12	Vesicular material	19.2	5.3
WB20	Wild boar^2^	2	Serum	17.81	5.3
WB20	Wild boar^2^	3	Serum	15.37	5.3
WB20	Wild boar^2^	3	Nasal swabs	27.04	neg
WB20	Wild boar^2^	3	Saliva	19.43	6
WB20	Wild boar^2^	4	Nasal swabs	25.32	5.7
WB20	Wild boar^2^	12	Vesicular material	24.4	7
WB25	Wild boar^2^	12	Vesicular material	23.84	5.7
P165	Pig^2^	12	Vesicular material	18.57	5.3
P165	Pig^2^	3	Saliva	27.12	6.7
P165	Pig^2^	3	Serum	18.63	5.3
P165	Pig^2^	4	Saliva	27.64	5.7
P161	Pig^2^	4	Saliva	28.31	7
P161	Pig^2^	4	Serum	15.73	3.7

Dpi, days post infection;

1 infected with serotype A22 Iraq 24/64;

2 infected with O Bulgaria 2011; neg, negative.

### Viruses

All FMDV reference strains used in this study were provided by the World Reference Laboratory for FMDV, Pirbright, UK. FMDV serotype A vaccinal strain (A Argentina 2001) was used to prepare the RNA molecular standard. The following FMDV strains were used for the assay validation: O Manisa, O BFS 1860, O TUR28/2011, O TUR33/2011, A24 Cruzeiro, A22 Iraq 24/64, A TUR 29/2011, A TUR64/2011, A BAR18/2011, C Oberbayern, C Noville, Asia1 Shamir, Asia TUR49/2011, Asia TUR51/2011, Asia TUR 65/2011, Asia PAK 5/2012, SAT1 Zimb22/89, SAT2 Egypt 2/2012, SAT2 Egypt 6/2012, SAT2 Libya 40/2012, and SAT3 Zim4/81. Also Swine vesicular disease virus (SVDV ITL/7/07, SVDV ITL/8/07) had been sent to the Friedrich-Loeffler-Institute by the Pirbright Institute, Pirbirght, UK. Classical swine fever virus (CSF) (Koslov genotype 1.1) was provided by the German national reference laboratory for CSF, Friedrich-Loeffler-Institute, Greifswald-Insel Riems, Germany.

### Generation of RNA Standard

The FMDV serotype A RNA was reverse transcribed and amplified using the QIAGEN OneStep RT-PCR kit (Qiagen, Hilden, Germany). The forward primer: 5′-CACTTCCACATGGATTATGGAACTG-3′, and the reverse primer: 5′-ACATCTGAGGGATTATGCGTCAC-3′ were used to amplify 260 nt of the highly conserved RNA polymerase (3D) gene of FMDV (7839–8098 nt of Genbank accession number JF749843) [13]. The RT-PCR reaction was set as follows: RT step 50°C/30 minutes, initial activation at 95°C/15 minutes, 30 cycles of 94°C/30 seconds, 58°C/60 seconds, and 72°C/60 seconds, and a final extension step of 72°C/10 minutes. The amplified fragment was ligated into pCR®II using the TA-cloning kit dual promoter with One shot® chemically competent *E.coli* (Invitrogen, Darmstadt, Germany). The ligated fragment was confirmed by sequencing (Seqlab, Goettingen, Germany) and the RNA was transcribed and quantified as previously described [18]. The standard was tested by a published real-time RT-PCR protocol [13] using the Light Cycler 2.0 and the LightCycler 480 RNA Master Hydrolysis Probes kit (Roche, Manheim, Germany).

### Real-time RT-RPA Primers and Exo-probes

**Figure 1:**
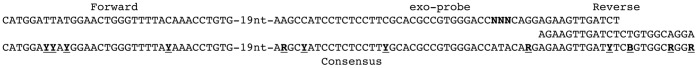
FMDV RT-RPA primers and exo-probe sequences aligned with the consensus sequence of 100 FMDV 3D genes downloaded from the Genbank. (Geneious® 6.1.5, Biomatters Limited, New Zealand). Mismatches are indicated in bold and underlined. The consensus sequence represents nt 7847–7961 of FMDV sequence JF749843. NNN are sites of the quencher and fluropohore in following order (BHQ1-dT) (Tetrahydrofuran) (FAM-dT). Y is C & T; R: A & G.

Nineteen forward primers, 20 reverse primers, and 4 exo-probes (Figure 1 and Figure S1 in File S1) were used to determine the combination yielding the highest RT-RPA assay sensitivity. They were designed using the available sequences of the FMDV 3D gene (Genbank accession numbers AF536538.1, GQ294636.1, EU400597.1, NC_004004.1, AY593830.1, DQ404158.1, EF552689.1) and were synthesized by TIB MOLBIOL (Berlin, Germany).

### RT-RPA Conditions

The FMDV RT-RPA was performed in the laboratory in a 50 µl volume using the TwistAmp™ exo lyophilized kit (TwistDx, Cambridge, UK) and addition of reverse transcriptase (RT) ‘Transcriptor’ (Roche, Mannheim, Germany). 420 nM RPA primers, 120 nM RPA exo-probe, 10 U RT ‘Transcriptor’ (Roche, Mannheim, Germany), 20 U RiboLock RNase inhibitor (Fisher, Schwerte, Germany), 2 µM DTT (Roche), 14 mM Mg acetate, 4x TwistAmp™ rehydration buffer (TwistDx), and 1 µl RNA template were added to the RPA strips containing a dried enzyme pellet as described [16]. Fluorescence detection in the FAM channel (excitation 470 nm and detection 520 nm) was performed in an ESEQuant tubescanner (Qiagen Lake Constance GmbH, Stockach, Germany) at 42°C for 20 minutes. A combined threshold and signal slope analysis confirmed by 2^nd^ derivative analysis offered by the tubescanner software was used for signal interpretation. In the field during the FMD 2012 outbreak in Egypt, the real-time RT-RPA assay was carried out using the TwistAmp™ RT exo (TwistDx, Cambridge, UK) according to the following formula: 420 nM RPA primers, 120 nM RPA exo-probe, 14 mM Mg acetate, 4x TwistAmp™ rehydration buffer (TwistDx), and 5 µl RNA template.

### Analytical Sensitivity

The analytical sensitivity of the RT-RPA assay was tested using a dilution range from 10^7^ to 10^1^ molecules/µl of the FMDV RNA standard in 8 replicates, the threshold time was plotted against molecules detected and a semi-log regression was calculated using Prism software (Graphpad Software Inc., San Diego, California). In addition, a probit regression was performed using the Statistica software (StatSoft, Hamburg, Germany).

### Specificity

The specificity of the FMDV RT-RPA assay was determined by testing twenty-one RNA preparations from the seven FMDV serotypes (listed above), and twelve FMDV-PCR-negative samples (saliva, serum, milk) from apparently healthy cows provided by the Animal Health Research Institute, Giza, Egypt. In addition, RNA of classical swine fever and swine vesicular disease viruses was screened for cross reactivity.

### Clinical Samples

Twenty-seven samples (Table 1) including vesicular material, saliva, serum, blood, and swabs from FMDV-immunization experiments performed at the Friedrich-Loeffler-Institute, Greifswald-Insel Riems, Germany were used to test the performance of the RT-RPA assay. The results were compared to real-time RT-PCR results.

A mobile RT-RPA unit was operated in Giza and Faiyum, Egypt between April 22nd–25th 2012 during the FMD outbreak. A total of 45 samples (heart, blood, serum, milk, saliva, and vesicular materials) from cattle, buffalo, and sheep were collected by the General Organization for Veterinary Services, Giza, Egypt according to the Egyptian Animal Welfare Act and all relevant institutional guidelines. The RNA was extracted using the Dynabeads® Silane viral nucleic acid (Invitrogen, Darmstadt, Germany) according to the manufacturer instructions. Then the FMDV RT-RPA was performed using the ready-to-use TwistAmp™ exo RT kits (TwistDx, Cambridge, UK) [16]. Real-time RT-PCR and FMDV serotyping of the samples was performed at the Animal Health Research Institute, Giza, Egypt as described [8]. In addition, the same samples were also tested by real-time RT-PCR and RT-RPA at the Virology Department in Goettingen. The R squared of the real–time RT-PCR cycle threshold and the real-time RT-RPA threshold time of clinical samples was calculated using linear regression analysis (Prism software, Graphpad Software Inc., San Diego, California).

### Real-time RT-PCR

At the Friedrich-Loeffler-Institute, Greifswald-Insel Riems, Germany, a Bio-Rad CFX 96 Real-Time Detection System was used in combination with the 3D-region assay [13], which amplifies a 88 bp fragment of the 3D region. The FMDV 3D real-time PCR assay was carried out as published except that the Superscript III One-Step RT system with Platinum Taq polymerase (Invitrogen, Darmstadt, Germany) was used. At the Virology Department, Goettingen, Germany, the same real-time RT-PCR assay was performed using the LightCycler 480 RNA Master Hydrolysis Probes kit on the Light Cycler 2.0. At the Animal Health Research Institute, Giza, Egypt, the test was performed as previously described [8].

## Results

### FMDV RT-RPA Assay Sensitivity and Specificity

**Figure 2:**
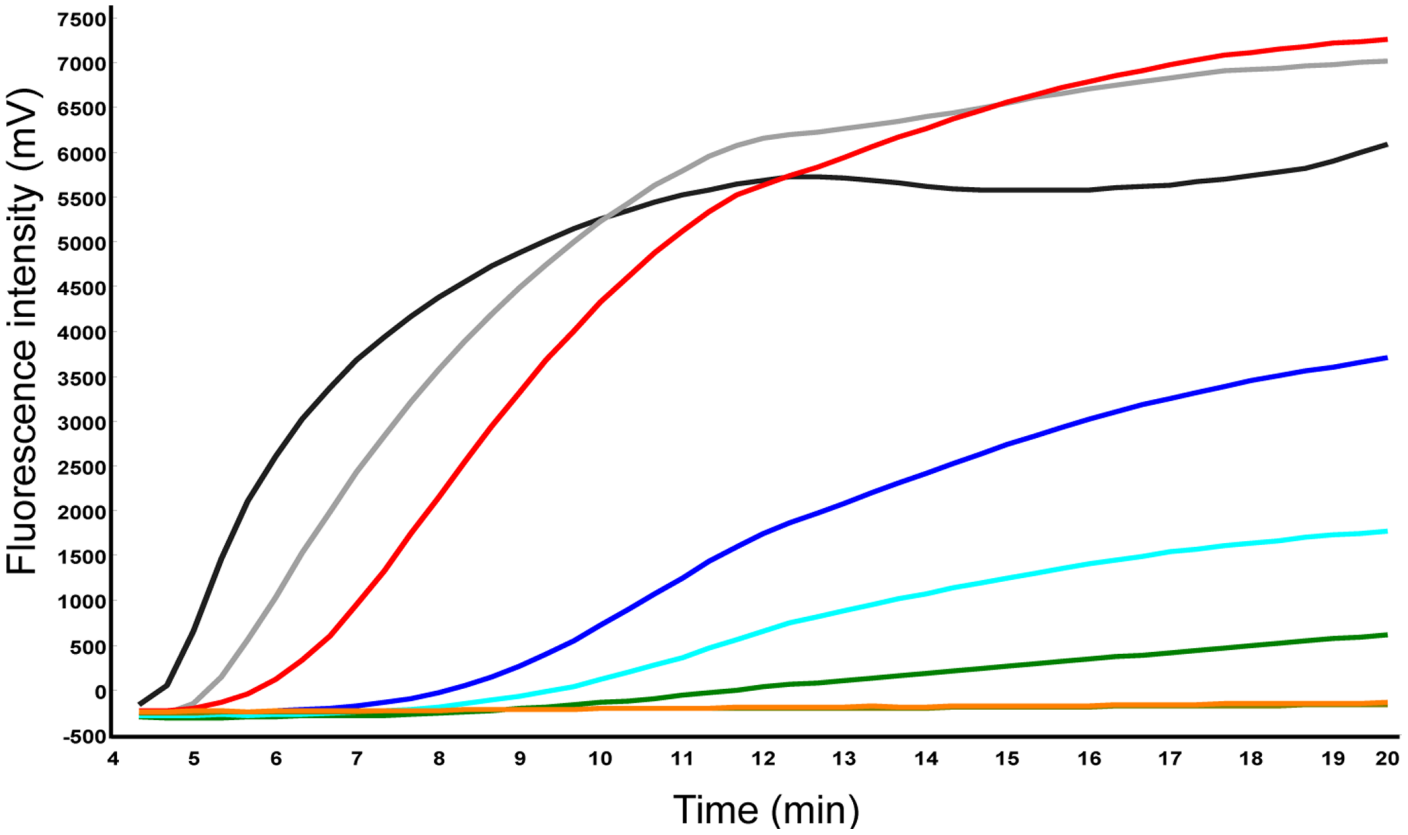
FMDV RT-RPA. Fluorescence development over time using a dilution range of 10^7^-10^1^ molecules/µl of the FMDV RNA standard (Graph generated by ESEquant tubescanner software). F04+R20+P2 were employed and the analytical sensitivity was 10^2^. 10^7^ represented by black line; 10^6^, gray; 10^5^, red; 10^4^, blue; 10^3^, green; 10^2^, cyan; 10^1^, dark khaki; negative control, orange.

**Figure 3:**
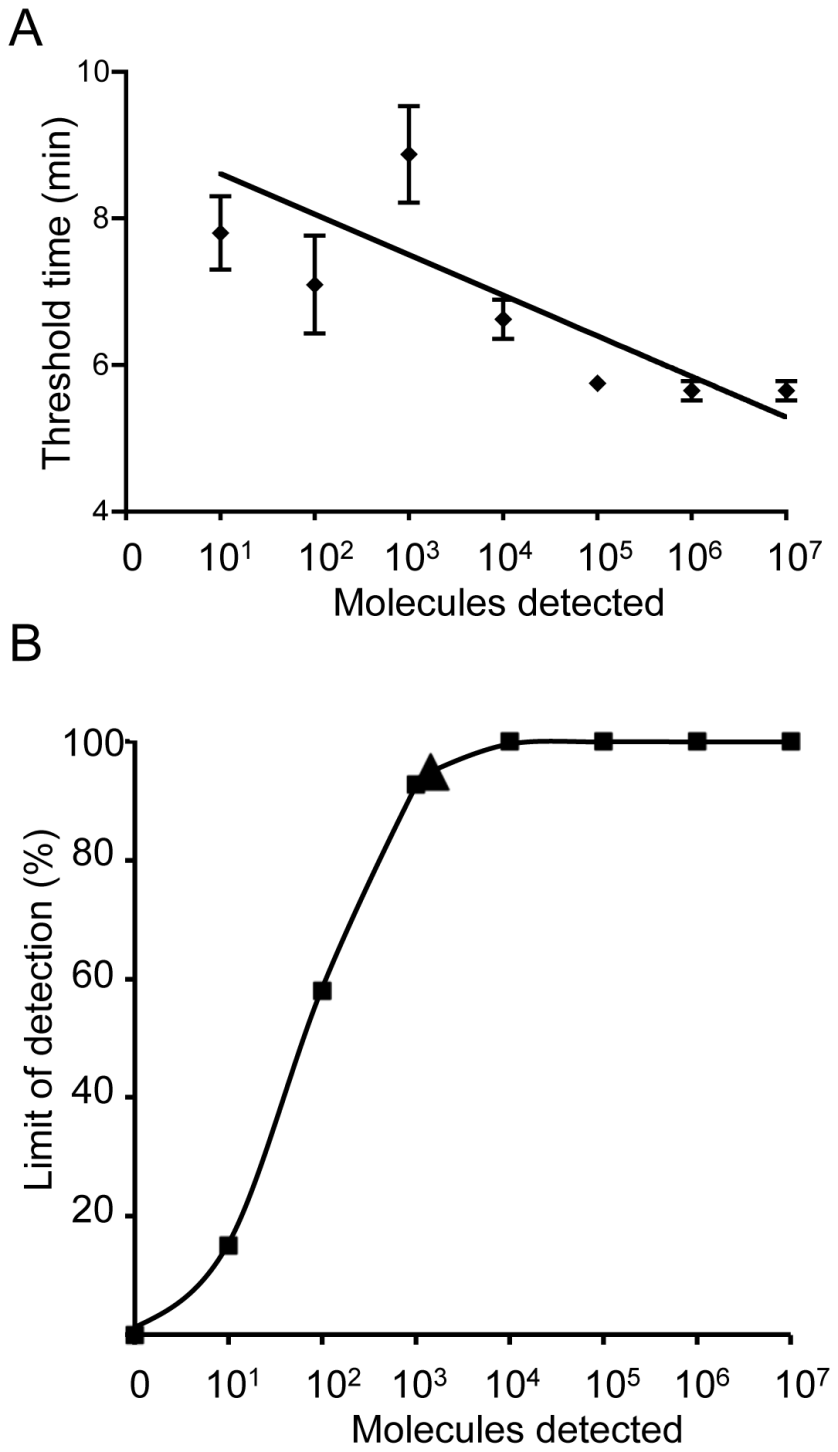
Performance and analytical sensitivity of the FMDV RT-RPA assay. A: Semi-logarithmic regression of the data collected from eight FMDV RT-RPA test runs on the RNA standard using Prism Software. It yielded results between 4–10 minutes. B: Probit regression analysis using Statistica software on data of the eight runs. The limit of detection at 95% probability (1436 RNA molecules) is depicted by a triangle.

An RNA standard representing 260 nt of the 3D gene of FMDV was produced via *in vitro* transcription from a plasmid containing a 3D gene fragment. A dilution range of 10^7^-10^1^ molecules/µl of the FMDV RNA standard was used to determine the analytical sensitivity of the RT-RPA assay in comparison to a real-time RT-PCR assay. To select a highly sensitive RT-RPA assay, combinations of forward primers (F), reverse primers (R), and exo-probes (P) listed in the Figure S1 in File S1 were tested. The sensitivity of most of the combinations was very low (e.g. 10^5^ molecules detected with F14+R19+P1). F04+R20+P2 yielded analytical sensitivity of 10^2^ RNA molecules detected (Figure 2) and was used for further assay validation. The time required to perform the assay to the limit of detection was 10 minutes (Figure 3A). Additionally, a probit regression analysis was performed using a data set of eight RT-RPA runs on the molecular RNA standard. The limit of detection in 95% of cases was 1436 RNA (Figure 3B). Interestingly, using an exo-probe designed for the reverse complementary strand (P2, Figure 1 & 2) yielded a better sensitivity than the positive sense exo-probe (P1, Figure S1 in File S1). In an attempt to shorten the exo-probes, P3, P4, synthesized with locked nucleic acid nucleotides (LNA) were tested but showed a very low sensitivity of 10^5^ RNA molecules detected (Figure S2A in File S1) and non-specific detection (Figure S2B in File S1), respectively. To confirm sensitivity of the RT-RPA assay, the RNA of twenty-one FMDV strains listed in material and methods part representing all serotypes were screened and all were detected (Figure S3 in File S1). In addition, unspecific amplification or detection was not observed on twelve FMDV-free samples from cows. No cross detections of the RNA of classical swine fever virus and swine vesicular disease virus was observed.

### Assay Performance on Clinical Samples

Twenty-seven samples encompassing vesicular materials, saliva, serum, blood, and swabs were collected from animals immunized with FMDV serotype A (A22 Iraq 24/64) and serotype O (Bulgaria 2011). The total RNA extracts of each sample were tested with both real-time RT-PCR and RT-RPA at the Friedrich-Loeffler-Institute, Greifswald-Insel Riems, Germany. In comparison to RT-PCR, the sensitivity of the RT-RPA assay was 74% (**n = 27**, Table 1). The real-time RT-PCR cycle threshold values for the false negative samples in RT-RPA ranged from 27.04 –31.1, and 36.14 (Table 1). Nevertheless, samples showing high cycle threshold values up to 39 in real-time RT-PCR (see below, Table S1 in File S1) indicating a very low molecular load were also scored positive by RT-RPA. The presence of relevant sequence variations was excluded because all FMDV strains were detected by RT-RPA. Therefore, the false negative results might be due to presence of inhibitors of the RT-RPA amplification and/or detection phase.

**Figure 4:**
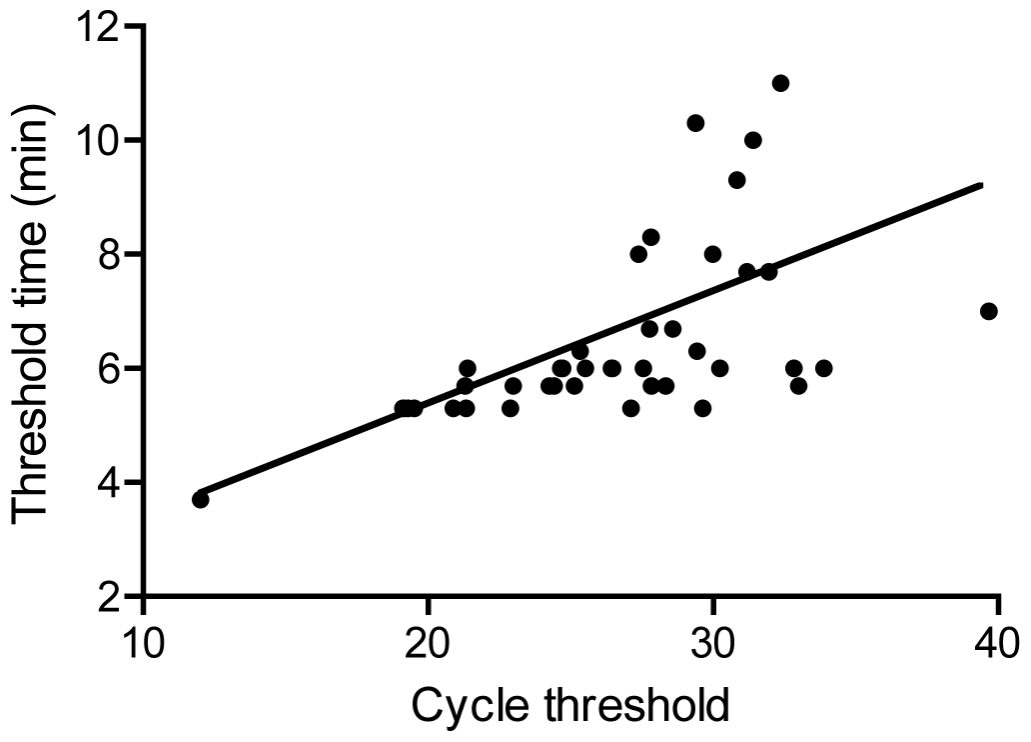
Comparison between real-time RT-RPA and RT-PCR for the detection of FMDV in clinical samples During Egypt 2012 FMD outbreak. Forty-five RNA extracts of samples collected from suspected cases of FMDV were screened. Linear regression analysis of RT-RPA threshold time (Y axis) and RT-PCR cycle threshold values (X axis) were determined by Prism software. R squared value was 0.26.

During the FMD outbreak in Egypt (spring 2012), a mobile RT-RPA unit was deployed. Forty-five samples were screened (Table S1 in File S1). The total RNA extracted from each sample was tested with real-time RT-PCR [8] (PCR-eg) and the RT-RPA assay using the RT exo kit (RPA-Twist) at the Animal Health Research Institute, Giza and the provincial laboratory Faiyum, Egypt. Inactivated RNA extracts were also tested with real-time RT-PCR [13] (PCR-de) and RT-RPA using the exo kits from Twist Dx™ in combination with RT ‘Transcriptor’ (Roche, Mannheim, Germany) (RPA-Roche) at the virology Department, Goettingen, Germany. The sensitivities of PCR-eg, PCR-de, RPA-Twist, and RPA-Roche were 89, 100, 62, and 98% (n = 45), respectively. RT-RPA using RT ‘Transcriptor’ was more sensitive than RT-RPA using the MulV based RT-RPA kit (Twist Dx™). A linear regression analysis of RPA-Roche threshold time and PCR-de cycle threshold was performed. No correlation was found between RT-PCR and RT-RPA values (R squared 0.26, Figure 4) but samples showing high cycle threshold values in real-time RT-PCR (e.g. 33.88 and 39.65, Table S1 in File S1) were also detected by RT-RPA. A linear regression analysis of the cycle threshold values of PCR-eg and PCR-de was calculated and R squared was 0.35 (Figure S4 in File S1).

## Discussion

Currently, molecular methods to detect FMDV are mostly used to confirm or rule out FMD in suspected cases. The only method routinely employed is RT-PCR, usually in the form of a real-time assay [13]. However, it is difficult to perform this test outside of a well-equipped laboratory as real-time cyclers and even mobile real-time cyclers are quite heavy, expensive, complex and must be operated by qualified staff. In addition, test run times are usually between 60–90 minutes. RT-PCR is therefore not suitable for routine point-of-care detection of FMDV. However, there clearly is a need for highly sensitive “pen-side” tests in the control of infectious animal diseases [4]. Laboratories may be overwhelmed by the number of samples in case of a large FMD epidemic. In any case, it will at least take several hours until the official veterinarian receives a laboratory result and thus may have to base his decision to cull a holding on clinical signs in suspected cases. Unless an FMD outbreak happens next to a well-equipped laboratory, only fast and highly sensitive point-of-care detection will make it feasible to cull confirmed FMDV positive animals in the incubation phase, i.e. before they can infect many other susceptible animals. In endemically infected countries, culling usually is not feasible, but highly sensitive point-of-care assays could contribute to FMD control by quickly providing a sound scientific basis for decisions on animal movement restrictions. In addition to confirm suspected clinical cases, it may make sense to check animals for FMDV at border control posts or on livestock markets. It is sometimes suggested to test also for persistent infection, but FMDV excretion by persistently infected animals is intermittent and thus even samples of optimal quality tested in a highly sensitive real-time RT-PCR will miss many carrier animals. Still, in some situations, e.g. if serological assays for antibodies to (infection-induced) non-structural proteins of FMDV can not resolve an unclear situation in a vaccinated population, herd testing by point-of-care assays may help to detect persistent infected animals. RT-RPA is carried out at a constant temperature (42°C) and results are produced in maximum 15 minutes, on a lightweight portable device (ESEQuant tubescanner). In this study an RT-RPA assay for the detection of FMDV was developed.

A FMDV molecular standard based on the 3D gene was used to determine the analytical sensitivity of the RT-RPA assay. Dozens of primers and exo-probes (Figure S1 in File S1) were tested to select an effective combination yielding a high sensitivity. Only the combination F04 (34 nt, GC content 41.1), R20 (22 nt, CG content 47.8) and P2 achieved a high analytical sensitivity (1436 RNA molecules detected, Figure 3B).

There are no clear-cut rules for the design of primers and exo-probe other than the advice mentioned on the Twist Dx website (http://www.twistdx.co.uk). Primers should be 30–35 nt in length, not contain multiple Gs in the first 5 bp of the 5′-end, and the GC content should be between 40–60%. Interestingly, R20, which yielded the highest RT-RPA sensitivity, is 22 nt in length, has two Gs at the 5′-end (Figure 1) and contains the lowest GC content of all reverse primers used in this study (47.8%, Table S2 in File S1). The RT-RPA primers were analyzed with Visual OMP (DNA software, MI, USA) for secondary structure and to determine the changes in the target secondary structure upon hybridization to primers [19]. All primers formed folded secondary structures (Figure S5 in File S1). *In silico* hybridization of the primers to the target sequence induced secondary structure changes of the target molecule (Figure S6 in File S1). Thus it appears that the successful combination of primers and exo-probe exerted an influence on the secondary structure of the target, which facilitated the RT-RPA amplification and detection process whereas the unsuccessful primers induced more complicated secondary structures not amenable to the RT-RPA reaction (see Figure S6 B and C in File S1).

According to exo-probe design rules, the exo-probe should be placed into the positive sense strand, and consist of at least 30 nt to the 5′ and 15 nt to the 3′ of the abasic site mimic. In contrast, P2, yielding a RT-RPA sensitivity increased by one log_10_-step, was designed complementary to the positive sense strand. It has 16 nt 5′ and 32 nt 3′ of the abasic site mimic and carries an inverse arrangement of fluorophore and quencher.

The detection step in RT-RPA depends on the separation between the fluorophore and its quencher via Exonuclease activity which releases the shorter sequence containing the quencher [15]. Then the shorter sequence containing the quencher is released and fluorescence is detected. In the approach recommended by Twist DX, the long 5′- part of the exo-probe and its non-protected 3′ end could influence amplification by acting as a primer in unwanted extension after release of the short 3′-part of the exo-probe. In contrast the short 16 nt 5′-part of P2 is removed leaving the hybridized long 32 nt phosphor group blocked 3′-part of P2, which therefore cannot be extended or otherwise interfere with further amplification. To improve RT-RPA design it might be necessary to consider this feature in exo-probe design in order to reduce interference of the hybridized exo-probe section with amplification. The same principle has been used in the design of real-time PCR, TaqMan probes, which are blocked at their 3′-end to avoid the consumption of reactions component in unfavourable probe extension [20].

The analytical sensitivity of the FMDV RT-RPA assay using F04, R20 and P2 was 1436 RNA molecules as determined by probit analysis of the results of eight assay runs (Figure 3B). The analytical sensitivity of the published real-time RT-PCR was 10 RNA molecules detected [13]. Nevertheless, the diagnostic sensitivity of the RT-RPA for the detection of FMDV during the outbreak in Egypt was 98% (Table S1 in File S1). The lowest titer of FMDV in the saliva of infected cows a few hours before onset of clinical signs ranges from 10^2^–10^3.75^ TCID_50_/ml [21]. As TCID_50_ readout depends on the presence of whole infective virions and the particle-to-infectivity ratio is 1000/1 [22], it follows that the detection limit of the RT-PPA assay is well in the range of the presence of FMDV RNA in the saliva of infected cows, but possibly not sufficient for virus detection in FMD carriers where RNA titer is very low [23]. The RT-RPA assay detected all seven FMDV serotypes, although primers and exo-probe cover an area containing some mismatches among the various subtypes (Figure 1). The maximum number of mismatches found within one sequence was five (e.g. accession numbers AY593782.1, HQ832584.1, and EU448374.1). In this case the length of primer and exo-probe compensates for mismatches in the target sequence, which usually cause real-time PCR small probes to fail or to lose sensitivity [24]. In a recent study, up to nine mismatches within RPA primers and exo-probe did not affect the HIV-1 RPA assay performance [25]. The RT-RPA assay did not detect RNA of other viruses causing vesicular diseases.

Recently, reverse transcription loop-mediated isothermal amplification (RT-LAMP) assays have been developed for rapid and sensitive detection of FMDV [26]–[28]. In contrast to RT-RPA, six primers are needed in RT-LAMP, which are difficult to design in a highly variant virus like FMDV. In addition, RT-LAMP results are produced within 45–60 minutes, while RT-RPA run time is limited to 4–10 minutes.

In conclusion, the RT-RPA assay was developed for rapid and sensitive identification of FMDV during outbreaks. Since RT-RPA reagents are available in a dry pellet form and a battery-charged portable instrument (ESEQuant tubescanner) can be used, the FMDV RT-RPA assay is suitable for mobile testing at border posts to monitor imported animals or for spot-of-infection screening in FMD outbreaks.

## Supporting Information

File S1 Figure S1, FMDV RT-RPA primers and probe sequences. Nineteen forward primers (F), 4 probes (P), and 20 reverse primers (R) were tested to select combinations yielding the highest analytical RPA sensitivity. NNN are sites of the quencher and fluropohore in following order (BHQ1-dT) (Tetrahydrofuran) (FAM-dT). LNA is probe containing locked nucleic acid (Bold and underlined). RC is the reverse complementary of the original sequence used in the experiment. Figure S2, The FMDV RT-RPA sensitivity with probes containing LNA nucleotide. Fluorescence development over time using a dilution range of 10^7^-10^1^ molecules/µl of the FMDV RNA standard (Graph generated by ESEquant tubescanner software). A: F04+R20+P3 were used for the amplification and detection steps and the sensitivity was 10^6^. 10^7^ represented by dot; 10^6^, box; 10^5^, triangular; 10^4^, diamond; 10^3^, star; 10^2^, vertical-line; 10^1^, horizontal-line; negative control, plane line. B: grey line is control negative with F04+R20+P4; black, F04+R20+P4+10^5^ of FMDV molecular standard; red, F04+R20+P2+10^5^ of FMDV molecular standard; blue, F04+R20+P3+10^5^ of FMDV molecular standard. Figure S3, The performance of the FMDV RT-RPA assay on RNA of serotypes O (Manisa, orange; BFS, dark khaki), SAT1 (SAT1 Zimb22/89, magenta), SAT2 (SAT2 Egypt 6/2012, cyan), C (C Oberbayern, black), and A (A22 Iraq 24/64, gray). Blue is the positive control (synthetic FMDV RNA) and orange is the negative control. Figure S4, Comparison between real-time PCR.eg and PCR.de for the detection of FMDV in clinical samples During Egypt 2012 FMD outbreak. Forty-five RNA extracts of samples collected from suspected cases of FMDV were screened. Linear regression analysis of cycle threshold (CT) values of PCR-eg (Y axis) and PCR-de (X axis) were determined by Prism software. R squared value was 0.35. Figure S5, Secondary structure of RPA primers. Structures were created by Visual OMP program ((DNA software, MI, USA). A, F02: B, F15; C, F04; D, R02; E, R06; F, R20. Figure S6, Primer hybridizing to the FMDV standard DNA affects its secondary structure. Structures were created by Visual OMP program ((DNA software, MI, USA). A, FMDV standard negative sense strand (7839–8098 of Genbank accession number JF749843) in unhybridized form: B, hybridized with F04; C, with F08; D, with R20. Primers are in black squares. Table S1, Detection of FMDV in samples from infected animals during the FMDV outbreak Egypt 2012 using real-time RT-PCR and RT-RPA. Table S2, GC content of the RPA forward and reverse primers.
(DOCX)
