# Supplementary material for: A Portable Reverse Transcription Recombinase Polymerase Amplification Assay for Rapid Detection of Foot-and-Mouth Disease Virus
Source: PLoS One. 2013 Aug 20;8(8):e71642. doi: 10.1371/journal.pone.0071642 (PMC3748043; doi:10.1371/journal.pone.0071642)
Supplement: File S1 — Figure S1, FMDV RT-RPA primers and probe sequences. Nineteen forward primers (F), 4 probes (P), and 20 reverse primers (R) were tested to select combinations yielding the highest analytical RPA sensitivity. NNN are sites of the quencher and fluropohore in following order (BHQ1-dT) (Tetrahydrofuran) (FAM-dT). LNA is probe containing locked nucleic acid (Bold and underlined). RC is the reverse complementary of the original sequence used in the experiment. Figure S2, The FMDV RT-RPA sensitivity with probes containing LNA nucleotide. Fluorescence development over time using a dilution range of 107-101 molecules/µl of the FMDV RNA standard (Graph generated by ESEquant tubescanner software). A: F04+R20+P3 were used for the amplification and detection steps and the sensitivity was 106. 107 represented by dot; 106, box; 105, triangular; 104, diamond; 103, star; 102, vertical-line; 101, horizontal-line; negative control, plane line. B: grey line is control negative with F04+R20+P4; black, F04+R20+P4+105 of FMDV molecular standard; red, F04+R20+P2+105 of FMDV molecular standard; blue, F04+R20+P3+105 of FMDV molecular standard. Figure S3, The performance of the FMDV RT-RPA assay on RNA of serotypes O (Manisa, orange; BFS, dark khaki), SAT1 (SAT1 Zimb22/89, magenta), SAT2 (SAT2 Egypt 6/2012, cyan), C (C Oberbayern, black), and A (A22 Iraq 24/64, gray). Blue is the positive control (synthetic FMDV RNA) and orange is the negative control. Figure S4, Comparison between real-time PCR.eg and PCR.de for the detection of FMDV in clinical samples During Egypt 2012 FMD outbreak. Forty-five RNA extracts of samples collected from suspected cases of FMDV were screened. Linear regression analysis of cycle threshold (CT) values of PCR-eg (Y axis) and PCR-de (X axis) were determined by Prism software. R squared value was 0.35. Figure S5, Secondary structure of RPA primers. Structures were created by Visual OMP program ((DNA software, MI, USA). A, F02: B, F15; C, F04; D, R02; E, R06; F, R20. F [file pone.0071642.s001.docx]

**Table S1 in file S1.** Detection of FMDV in samples from infected animals during the FMDV outbreak Egypt 2012 using real-time RT-PCR and RT-RPA.

| **No.** | **Sample Code** | **Species** | **Sample Type** | **Serotype**  **(By sequencing)** | **PCR-eg** | **PCR-de** | **RPA-Twist** | **RPA- Roche** |
| --- | --- | --- | --- | --- | --- | --- | --- | --- |
|  |  |  |  |  | **CT** | **CT** | **min** | **min** |
| 1 | C2 | CATTLE | S | SAT2 | 28.36 | 22.98 | 5.3 | 5.7 |
| 2 | C3 | CATTLE | E | SAT2 | 23.23 | 28.33 | 5.7 | 5.7 |
| 3 | C4 | CATTLE | E | SAT2 | 23.66 | 19.16 | 5.3 | 5.3 |
| 4 | C5 | CATTLE | B | NA | 25.89 | 19.5 | 5.3 | 5.3 |
| 5 | C6 | BUFFALOE | S | SAT2 | 35.85 | 32.36 | Neg | 11 |
| 6 | C7 | CATTLE | H | SAT2 | 21.72 | 20.88 | 5.3 | 5.3 |
| 7 | C8 | CATTLE | E | SAT2 | 25.32 | 22.87 | 5.3 | 5.3 |
| 8 | C9 | CATTLE | S | SAT2 | 31.32 | 27.81 | 6 | 8.3 |
| 9 | C10 | CATTLE | H | SAT2 | 23.73 | 19.3 | 5.3 | 5.3 |
| 10 | C11 | CATTLE | E | SAT2 | 19.28 | 12 | 3.7 | 3.7 |
| 11 | C12 | CATTLE | E | A | 18.27 | 19.1 | 5.3 | 5.3 |
| 12 | C13 | CATTLE | E | SAT2 | 26.85 | 27.1 | 6 | 5.3 |
| 13 | C14 | CATTLE | E | SAT2 | 35.72 | 24.24 | 6 | 5.7 |
| 14 | C15 | CATTLE | E | SAT2 | 27.56 | 24.702 | 9.3 | 6 |
| 15 | C16 | CATTLE | E | SAT2 | 31.41 | 25.13 | 6.7 | 5.7 |
| 16 | B1 | CATTLE | E | NA | 39.17 | 31.39 | Neg | 10 |
| 17 | B2 | CATTLE | E | NA | Neg | 31.97 | Neg | Neg |
| 18 | B3 | CATTLE | E | SAT2 | 27.76 | 24.41 | 5.3 | 5.7 |
| 19 | B4 | CATTLE | S | SAT2 | Neg | 28.57 | Neg | 6.7 |
| 20 | B5 | SHEEP | S | O | Neg | 26.43 | 9 | 6 |
| 21 | B6 | CATTLE | H | SAT2 | 32.4 | 27.38 | 9.7 | 8 |
| 22 | B7 | BUFFALOE | H | SAT2 | Neg | 27.82 | 6.3 | 5.7 |
| 23 | B8 | BUFFALOE | E | NA | 39 | 27.76 | 9 | 6.7 |
| 24 | B9 | CATTLE | S | SAT2 | 23.21 | 21.3 | 5.3 | 5.7 |
| 25 | B10 | BUFFALOE | E | SAT2 | 33.34 | 25.32 | Neg | 6.3 |
| 26 | B11 | BUFFALOE | S | NEG | 34.68 | 31.15 | Neg | 12.7 |
| 27 | B12 | BUFFALOE | S | SAT2 | 34.19 | 29.37 | Neg | 10.3 |
| 28 | B13 | CATTLE | E | SAT2 | 32.75 | 27.88 | Neg | 12.3 |
| 29 | B14 | BUFFALOE | E | SAT2 | 24.6 | 24.65 | 5.7 | 6 |
| 30 | B15 | CATTLE | E | POS | 31.75 | 29.43 | Neg | 6.3 |
| 31 | B16 | CATTLE | S | SAT2 | 30.89 | 31.94 | Neg | 7.7 |
| 32 | F1 | CATTLE | E | N/A | 22.6 | 32.99 | Neg | 5.7 |
| 33 | F2 | CATTLE | E | N/A | 29.5 | 27.55 | Neg | 6 |
| 34 | F3 | CATTLE | S | N/A | 29.9 | 21.37 | 6 | 6 |
| 35 | F4 | CATTLE | B | N/A | 33.8 | 21.32 | 6 | 5.3 |
| 36 | F5 | CATTLE | B | N/A | 31 | 26.45 | 6 | 6 |
| 37 | F6 | CATTLE | B | N/A | 31.7 | 25.5 | 6 | 6 |
| 38 | F7 | CATTLE | B | N/A | 20.4 | 29.62 | 6 | 5.3 |
| 39 | F8 | CATTLE | S | N/A | 42.5 | 30.22 | Neg | 6 |
| 40 | F9 | CATTLE | S | N/A | 32.6 | 33.88 | Neg | 6 |
| 41 | F10 | CATTLE | M | N/A | 34.78 | 32.82 | Neg | 6 |
| 42 | F11 | CATTLE | M | N/A | 35.33 | 39.65 | Neg | 7 |
| 43 | F12 | CATTLE | M | N/A | Neg | 31.17 | Neg | 7.7 |
| 44 | F14 | CATTLE | B | N/A | 35.64 | 30.82 | 8 | 9.3 |
| 45 | F15 | CATTLE | B | N/A | 35.33 | 29.98 | 9.7 | 8 |

E, vesicular material; S, saliva; H, heart: B, blood; M, milk; Neg, negative; N/A, not applicable; PCR-eg, RT-PCR as described [[8](#_ENREF_8)]; RPA-Twist, RT-RPA using RT exo kits from Twist Dx™ (TwistDx, Cambridge, UK); PCR-de, RT-PCR as described [[12](#_ENREF_12)]; RPA-Roche, RT-RPA with exo kits from Twist Dx^TM^ but using RT from Roche (Roche, Mannheim, Germany); CT, cycle threshold

**Table S2 in file S1.** GC content of the RPA forward and reverse primers.

| **Name** | **AT content** | **GC content** | **GC%** | **GC at 5´** | **RPA sensitivity** |
| --- | --- | --- | --- | --- | --- |
| F01 | 16 | 13 | 44.8 | 2 | N/A |
| F02 | 20 | 13 | 39.3 | 2 | N/A |
| F03 | 18 | 12 | 40 | 2 | N/A |
| F04 | 20 | 14 | 41.1 | 3 | 1.00E+02 |
| F05 | 16 | 14 | 46.6 | 2 | N/A |
| F06 | 16 | 14 | 46.6 | 2 | N/A |
| F07 | 15 | 15 | 50 | 3 | N/A |
| F08 | 17 | 13 | 43.3 | 2 | N/A |
| F09 | 19 | 11 | 36.6 | 1 | N/A |
| F10 | 19 | 11 | 36.6 | 0 | N/A |
| F11 | 17 | 13 | 40 | 1 | N/A |
| F12 | 20 | 12 | 39.9 | 2 | N/A |
| F13 | 21 | 13 | 38.2 | 2 | N/A |
| F14 | 21 | 14 | 40 | 3 | 1.00E+03 |
| F15 | 22 | 14 | 38.8 | 2 | 1.00E+2 -1.00E+03 |
| F16 | 17 | 11 | 39.2 | 2 | N/A |
| F17 | 16 | 11 | 40.7 | 2 | N/A |
| F18 | 15 | 11 | 42.3 | 2 | N/A |
| F19 | 14 | 9 | 39.1 | 2 | N/A |
| R01 | 13 | 19 | 59.3 | 2 | N/A |
| R02 | 14 | 17 | 54.8 | 3 | N/A |
| R03 | 14 | 19 | 57.5 | 1 | N/A |
| R04 | 12 | 22 | 64.7 | 3 | N/A |
| R05 | 12 | 23 | 65.7 | 3 | N/A |
| R06 | 13 | 18 | 58 | 4 | N/A |
| R07 | 14 | 17 | 54.8 | 4 | 1.00E+03 |
| R08 | 13 | 18 | 58 | 4 | N/A |
| R09 | 12 | 19 | 61.2 | 3 | 1.00E+03 |
| R10 | 11 | 20 | 64.5 | 4 | N/A |
| R11 | 11 | 20 | 64.5 | 3 | N/A |
| R12 | 14 | 18 | 56.2 | 4 | N/A |
| R13 | 13 | 17 | 56.6 | 5 | N/A |
| R14 | 13 | 16 | 55.1 | 4 | N/A |
| R15 | 13 | 15 | 53.5 | 4 | 1.00E+03 |
| R16 | 13 | 14 | 51.8 | 3 | N/A |
| R17 | 13 | 13 | 50 | 3 | N/A |
| R18 | 13 | 12 | 48 | 3 | N/A |
| R19 | 12 | 12 | 50 | 3 | 1.00E+03 |
| R20 | 12 | 11 | 47.8 | 3 | 1.00E+02 |

F, forward primer; R, reverse primer; N/A, not applicable

**
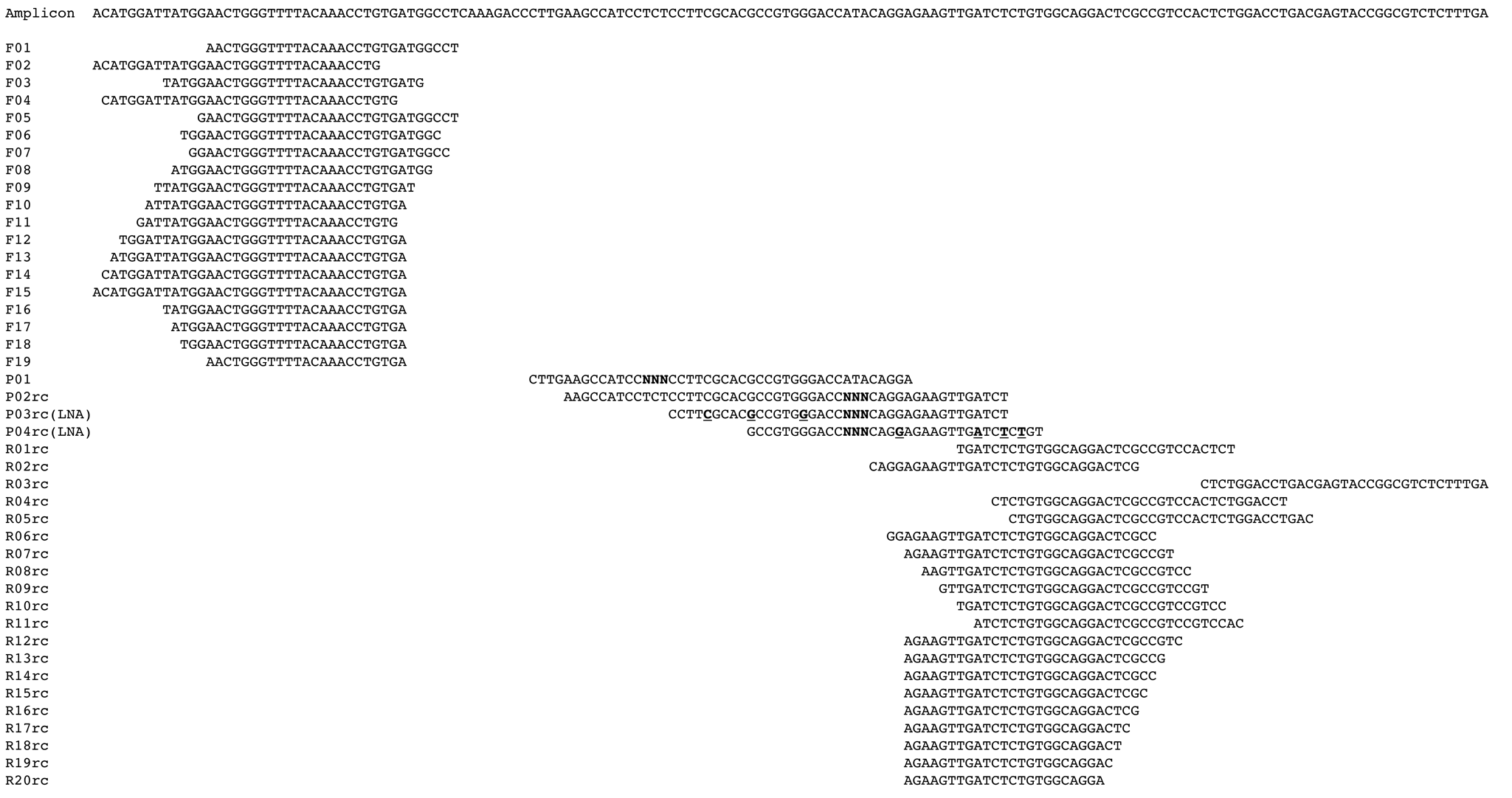
**

**Figure S1 in file S1. FMDV RT-RPA primers and probe sequences.** Nineteen forward primers (F), 4 probes (P), and 20 reverse primers (R) were tested to select combinations yielding the highest analytical RPA sensitivity. NNN are sites of the quencher and fluropohore in following order (BHQ1-dT) (Tetrahydrofuran) (FAM-dT). LNA is probe containing locked nucleic acid (Bold and underlined). RC is the reverse complementary of the original sequence used in the experiment.

**
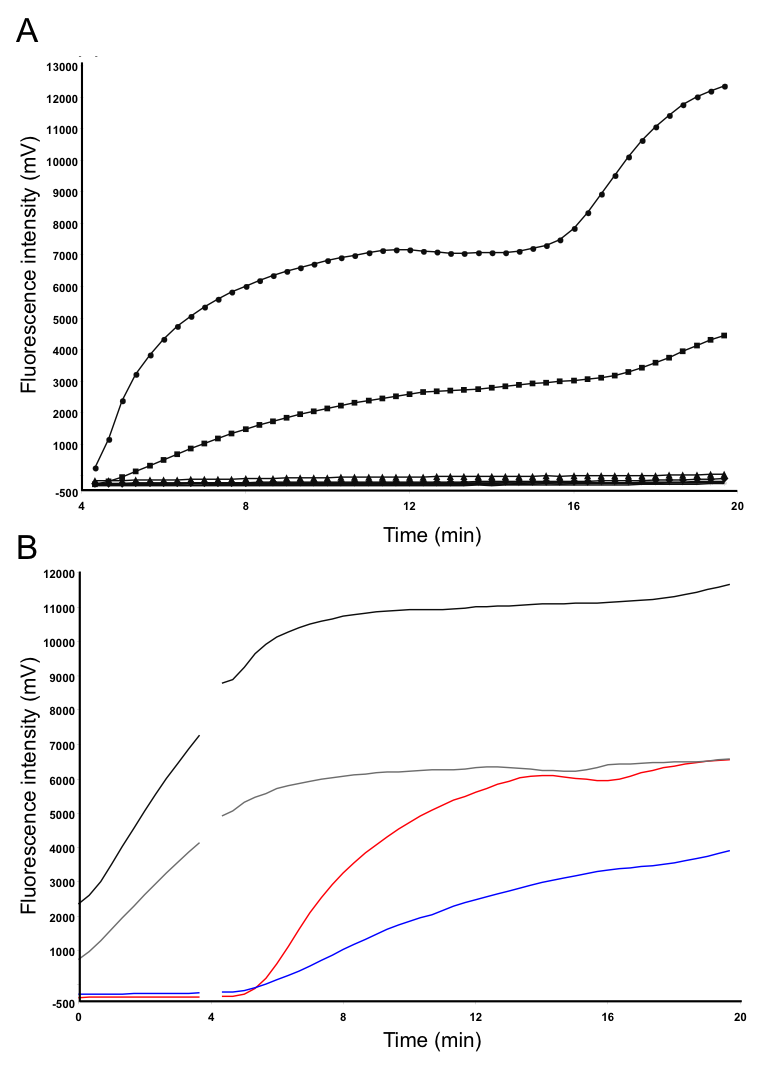
**

**Figure S2 in file S1. The FMDV RT-RPA sensitivity with probes containing LNA nucleotide.** Fluorescence development over time using a dilution range of 10^7^-10^1^ molecules/µl of the FMDV RNA standard (Graph generated by ESEquant tubescanner software). A: F04+R20+P3 were used for the amplification and detection steps and the sensitivity was 10^6^. 10^7^ represented by dot; 10^6^, box; 10^5^, triangular; 10^4^, diamond; 10^3^, star; 10^2^, vertical-line; 10^1^, horizontal-line; negative control, plane line. B: grey line is control negative with F04+R20+P4; black, F04+R20+P4+10^5^ of FMDV molecular standard; red, F04+R20+P2+10^5^ of FMDV molecular standard; blue, F04+R20+P3+10^5^ of FMDV molecular standard.


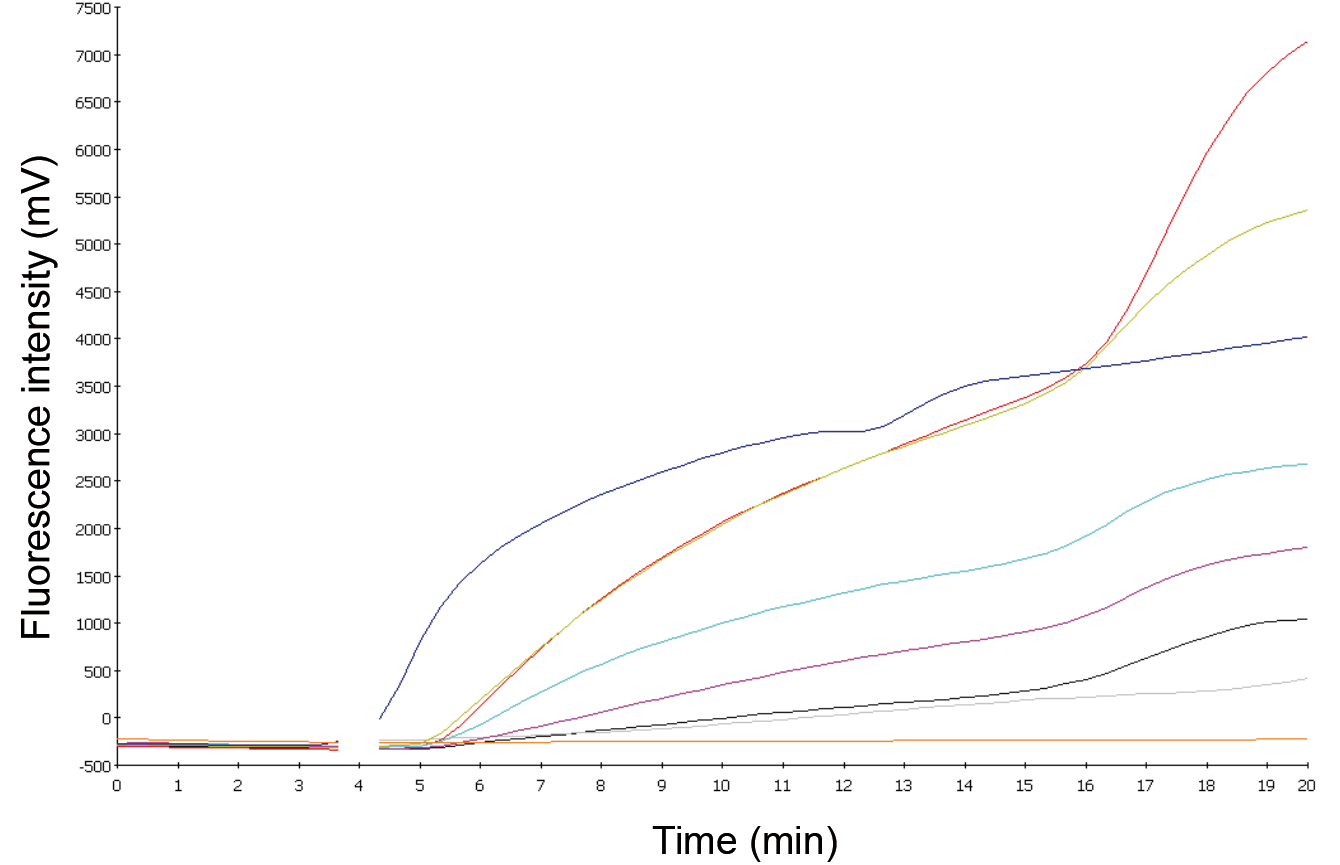


**Figure S3 in file S1. The performance of the FMDV RT-RPA assay on RNA of serotypes O (Manisa, orange; BFS, dark khaki), SAT1 (SAT1 Zimb22/89, magenta), SAT2 (SAT2 Egypt 6/2012, cyan), C (C Oberbayern, black), and A (A22 Iraq 24/64, gray). Blue is the positive control (synthetic FMDV RNA) and orange is the negative control.**


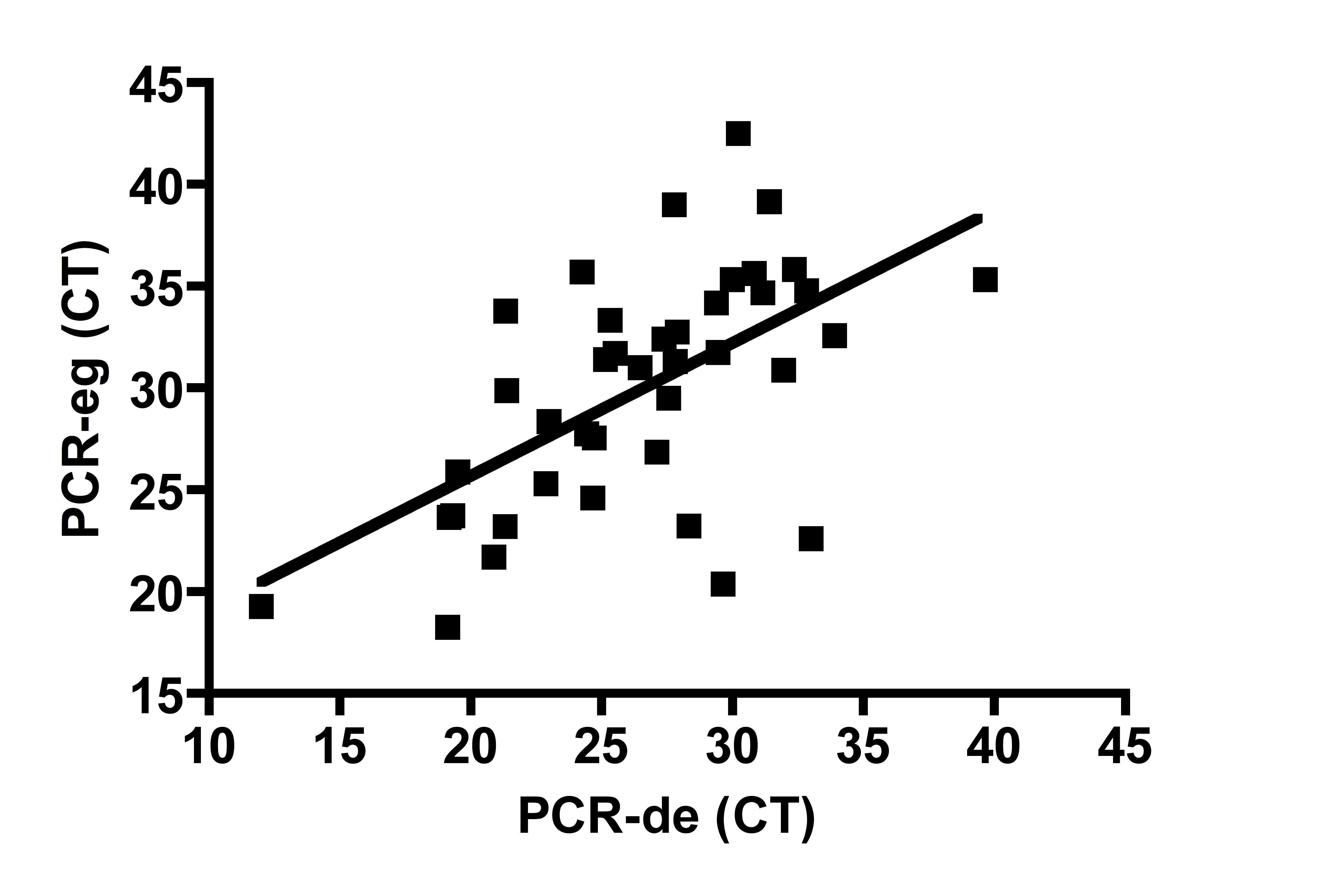


**Figure S4 in file S1. Comparison between real-time PCR.eg and PCR.de for the detection of FMDV in clinical samples During Egypt 2012 FMD outbreak.** Forty-five RNA extracts of samples collected from suspected cases of FMDV were screened. Linear regression analysis of cycle threshold (CT) values of PCR-eg (Y axis) and PCR-de (X axis) were determined by Prism software. R squared value was 0.35.


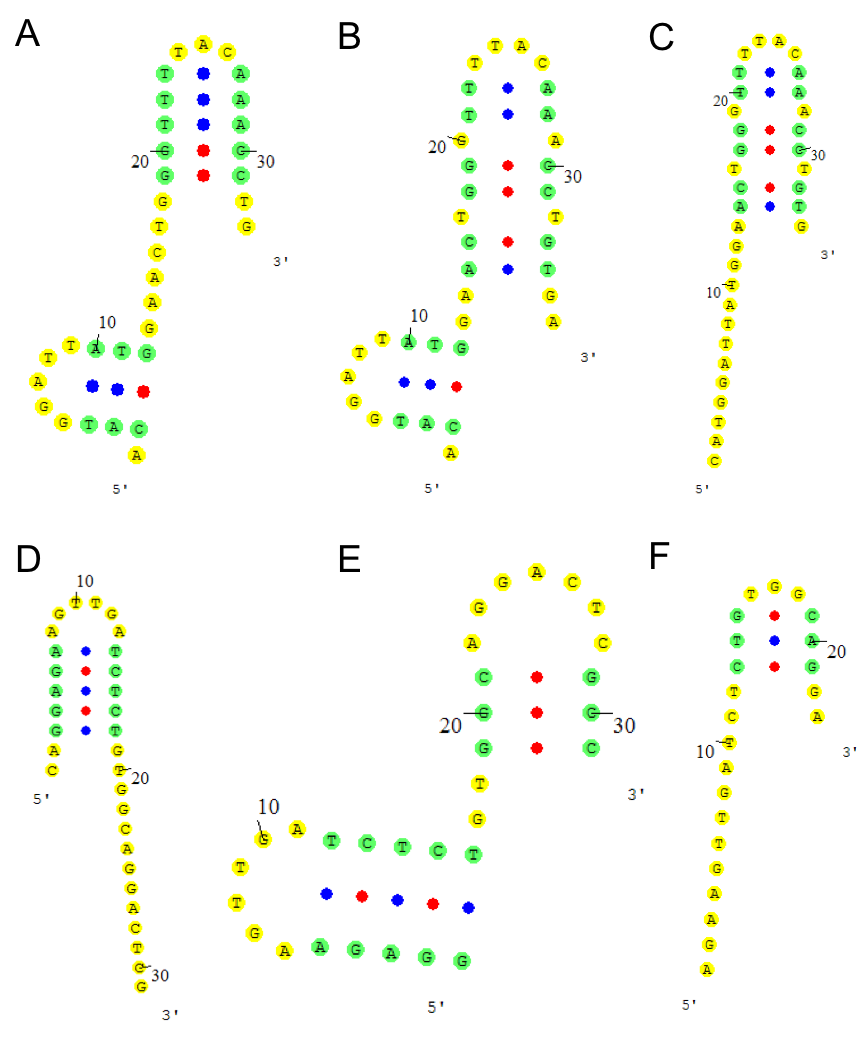


**Figure S5 in file S1. Secondary structure of RPA primers.** Structures were created by Visual OMP program ((DNA software, MI, USA). A, F02: B, F15; C, F04; D, R02; E, R06; F, R20


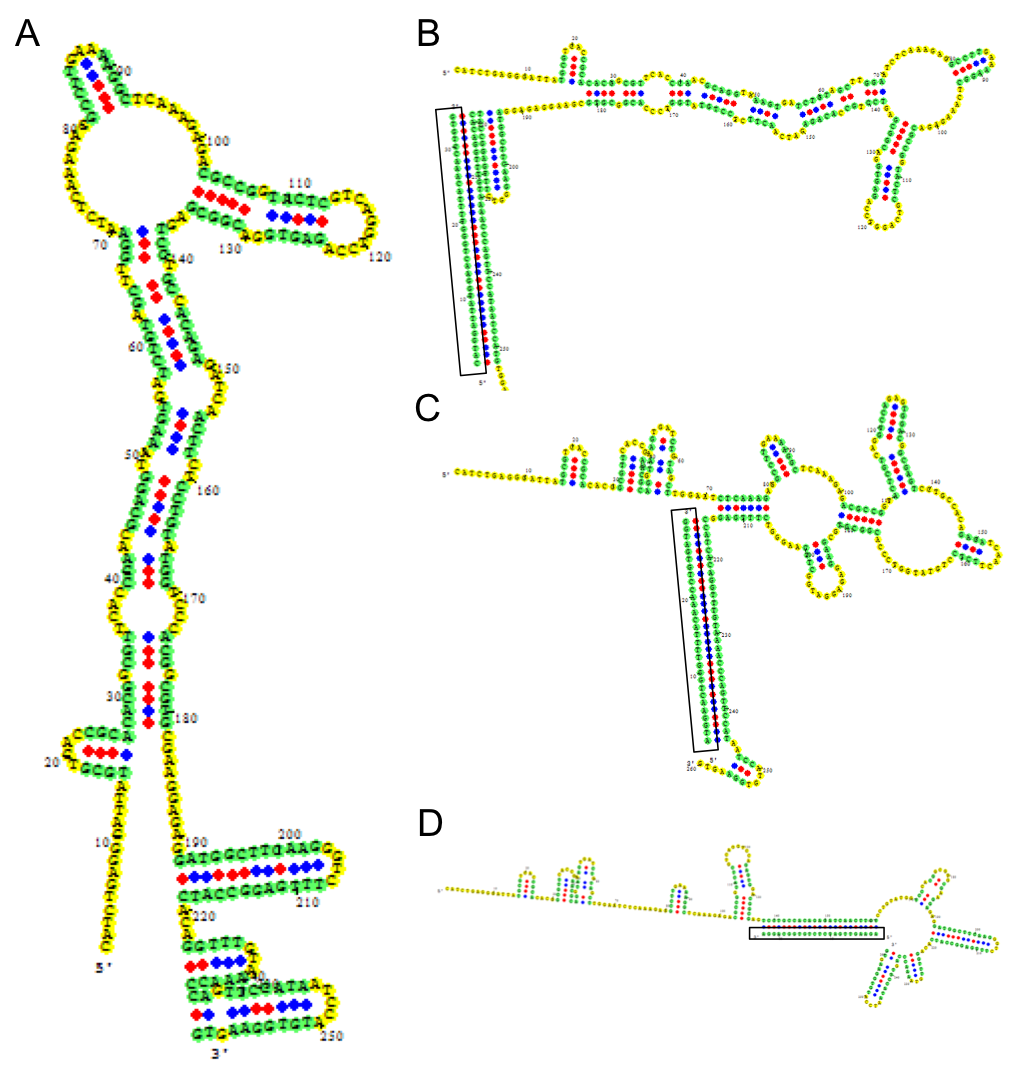


**Figure S6 in file S1. Primer hybridizing to the FMDV standard DNA affects its secondary structure.** Structures were created by Visual OMP program ((DNA software, MI, USA). A, FMDV standard negative sense strand (7839-8098 of Genbank accession number JF749843) in unhybridized form: B, hybridized with F04; C, with F08; D, with R20. Primers are in black squares.
